# Investigation of occupational accidents in Brazil: a decade-long ecological study (2012-2022)

**DOI:** 10.47626/1679-4435-2024-1289

**Published:** 2025-01-16

**Authors:** Raissa Sucar Pereira de Araújo

**Affiliations:** 1 Medicine, Universidade Potiguar, Natal, RN, Brazil.

**Keywords:** public health, surveillance of the workers health, accidents, occupational, occupational accidents registry, saúde pública, vigilância em saúde do trabalhador, acidentes de trabalho, notificação de acidentes de trabalho

## Abstract

**Introduction:**

This study conducted a time trend analysis of occupational accidents in Brazil from 2012 to 2022.

**Objectives:**

To contribute to more effective strategies for controlling the associated risks.

**Methods:**

Using an ecological, retrospective, descriptive, quantitative approach, public data were collected using the TABNET application from Brazil Unified Health System Department of Information. The collection covered all reported cases of occupational accidents among Brazilian residents in December 2023. The study focused on inequalities according to educational levels, age group, education level, and regions, while also investigating health care and the evolution of cases.

**Results:**

The results revealed a concerning increase in notifications, reaching 301,107 cases in 2022; an increase not only in quantity, but also in the severity of accidents.

**Conclusions:**

The method used provided a comprehensive and detailed analysis, allowing educational and regional disparities to be identified, and highlighted the importance of seeking health care. However, the gap in documentation highlights a need to improve data collection for a more complete grasp of the impacts of occupational accidents, guiding effective interventions to promote occupational safety.

## Abstract (pt)

**Introdução:**

Este estudo realizou uma análise da tendência temporal dos acidentes de trabalho no Brasil de 2012 a 2022.

**Objetivos:**

Contribuir para estratégias mais eficazes de controle dos riscos associados.

**Métodos:**

Utilizando uma abordagem ecológica, retrospectiva, descritiva e quantitativa, foram coletados dados públicos acessíveis pelo aplicativo TABNET, do Departamento de Informática do Sistema Único de Saúde. A coleta abrangeu todos os casos notificados de acidentes de trabalho entre residentes do território brasileiro, realizada em dezembro de 2023. O estudo enfocou desigualdades segundo níveis educacionais, faixa etária, escolaridade e regiões, além de investigar o atendimento médico e a evolução dos casos.

**Resultados:**

Os resultados revelaram um aumento preocupante nas notificações, atingindo 301.107 casos em 2022, com crescimento não apenas quantitativo, mas também na gravidade dos acidentes.

**Conclusões:**

A metodologia utilizada proporcionou uma análise abrangente e detalhada, permitindo identificar disparidades educacionais e regionais, bem como evidenciar a importância da busca por assistência médica. Contudo, a lacuna na documentação destaca a necessidade de aprimorar a coleta de dados para uma compreensão mais completa dos impactos dos acidentes de trabalho, orientando intervenções eficazes na promoção da segurança ocupacional.

## INTRODUCTION

Occupational accidents are injuries that result from work and are defined by law. Article 19 of Law No. 8,213/1991, which regulates the Plano de Benefícios da Previdência Social (Brazil Social Security Benefits Plan), defines occupational accidents as those that “occur in the course of work on behalf of the company or as a result of the work of insured persons.” Importantly, to be considered an occupational accident or disease, it is essential that Instituto Nacional do Seguro Social (INSS, Brazil National Social Security Institute) medical expert declares it to be so, technically establishing the causal relationship between the incident and the injury, the illness and work, or the cause of death and the accident.^1^

The Ministério da Saúde (Ministry of Health) Política Nacional de Saúde do Trabalhador e da Trabalhadora (PNSTT, Brazil National Workers’ Health Policy) aims to define, in article 2, the principles, guidelines, and strategies to be followed by the three government levels of the Sistema Único de Saúde (SUS, Brazil Unified Health System).^2^ The main objective is to promote comprehensive health care for workers, particularly in terms of health surveillance, promotion, and protection. The PNSTT directs efforts towards preventing work-related morbidity and mortality, ensuring decent and safe conditions for workers, and helping to improve quality of life and well-being in the workplace.

The Rede Nacional de Atenção Integral à Saúde do Trabalhador (RENAST, Brazil National Network for Comprehensive Workers’ Health Care), together with the Centros Estaduais e Regionais de Referência em Saúde do Trabalhador (CERESTs, State and Regional Workers’ Health Reference Centers), plays a crucial role in promoting occupational health in Brazil.^3^ CERESTs, as part of RENAST, act as technical and scientific support centers aimed at preventing and monitoring working conditions, diagnosing and treating diseases associated with the workplace.^4^ Additionally, these centers are responsible for the mandatory notification of work-related diseases and illnesses, as determined by the Sistema de Informação de Agravos de Notificação (SINAN, Brazil Notifiable Disease Surveillance System).^5^

SINAN is a key system for collecting and analyzing epidemiological data, including cases of occupational diseases recorded by CERESTs. Notification is mandatory according to article 8 of Law No. 6,259 of October 30, 1975, and is incumbent on physicians, other health professionals, and managers of public and private health services. This registry ensures that cases of work-related injuries are properly recorded and treated, contributing to safe and healthy workplaces.^6^

From April 2007, the INSS implemented a new approach to granting accident compensation benefits, resulting in significant improvements in the statistics relating to occupational accidents. This initiative aimed to mitigate the underreporting of occupational accidents and illnesses. Studies based on statistical and epidemiological principles, involving a joint analysis of the International Classification of Diseases (ICD-10) and the Classificação Nacional de Atividades Econômicas (CNAE, Brazil National Classification of Economic Activities) codes, enabled the identification of associations between health problems and work. This process eventually resulted in the construction of an association matrix between the CNAE and ICD-10 codes, called the Nexo Técnico Epidemiológico Previdenciário (NTEP, Brazil Social Security Epidemiological Technical Nexus). The NTEP has become a valuable complementary tool for INSS medical examinations, helping to analyze and conclude work disability. This approach represented a significant improvement in the reporting and understanding of occupational accidents. However, underreporting is still a problem in Brazil.^7^

According to the International Labor Organization (ILO),^8^ the report “Joint Estimates of the Work-related Burden of Disease and Injury, 2000-2016: Global Monitoring Report” highlights that almost 2 million people die from work-related causes every year. This reflects a need for improvements in health and safety in the workplace.

Health education is of major importance in preventing occupational accidents, as it enables workers to update their understanding and improve their technical, scientific, and behavioral skills when performing their professional activities. This approach helps to optimize individual and collective performance, and fosters human, professional, and institutional development.

In this context, it is worth mentioning the Programa Nacional de Educação em Segurança e Saúde no Trabalho (PROEDUC, Brazil National Program for Occupational Health and Safety Education). This program is funded by the federal government through Fundacentro and aims to promote educational initiatives through courses aimed at learning about safety and health-related issues in the workplace. It operates along four fronts: 1) the implementation of pedagogical practices in Segurança e Saúde do Trabalhador (Worker Safety and Health), using the Pedagogia dos Projetos de Trabalho (Work Project Pedagogy); 2) training and updating in Saúde e Segurança no Trabalho (SST, Work Health and Safety); 3) the integration of SST into formal education, exploring approaches such as learning with nature and Educação de Jovens e Adultos (EJA, Youth and Adult Education); and 4) the development of research into Práticas Pedagógicas (Pedagogy Practices) on SST, health, work, sustainable development in the transport sector, and work and subjectivity in marble quarries.^9^

Occupational accidents have emerged as a significant public health concern in Brazil.^10^ Not only does this phenomenon cause losses for both workers and employers, but it also has a negative impact on Brazilian economy. In view of this, it is imperative to thoroughly analyze its various aspects, with a view to gaining a more complete and effective understanding, and implementing effective strategies to control the associated risks.^11^

This study aims to conduct a time trend analysis of occupational accidents in Brazil, per region, from 2012 to 2022. Therefore, the number of accidents according to the level of education and age group of professionals, and also the evolution of reported cases and the presence or absence of health care were investigated.

## METHODS

This is an ecological study with a retrospective, descriptive and quantitative approach, which analyzed public data accessed through the TABNET application of the Departamento de Informática do SUS (DATASUS, SUS Information Technology Department). The source used is from the Ministério da Previdência Social (MPS, Brazil Ministry of Social Security) and was registered on SINAN Data collection were conducted in December 2023, covering all reported cases of occupational accidents involving residents in Brazil from 2012 to 2022. The use of secondary data meant that the study was not submitted to the Research Ethics Committee.

The variables were organized in tables using MS Excel and presented in absolute and relative numbers. The variables included notifications per year (2012-2022), types of disability (temporary partial disability, temporary total disability, permanent partial disability, and permanent total disability), outcomes (death as a result of the accident, cure), level of education (illiterate, primary education, secondary education, higher education, not applicable), regions of notification (North, Northeast, Southeast, South, Midwest) and health care (missing/blank, yes, no). Additionally, the mortality rate was calculated per year by dividing the number of deaths caused by occupational accidents by the total number of notifications and multiplying the result by 100.

Finally, to support the data obtained from SINAN, searches were conducted on the PubMed and SciELO platforms, using the descriptors “labor accidents” and “Brazil”, connected by the Boolean operator “AND.” A total of 84 publications were found on SciELO and 206 on PubMed. The inclusion criteria used to filter out the articles relevant to this study were complete articles published in the last 5 years. The exclusion criteria included theses, dissertations, incomplete articles, or articles not related to the topic. In addition, work safety books and reliable public domain data were consulted to support the discussion of the results. This strategy allowed for a more robust and contextualized analysis, enriching the understanding of the data collected and placing it within the broader context of existing research and scientific publications on occupational accidents in Brazil.

## RESULTS AND DISCUSSION

Table 1 shows that notifications of occupational accidents have risen substantially over the years, revealing a concerning trend in the Brazilian labor scenario. In 2012, 75,481 notifications were recorded, a figure that grew steadily in subsequent years. In 2022, notifications reached a staggering record of 301,107, a significant increase compared to previous years. This growth can be attributed to various factors, such as changing working conditions, industrial expansion, or even greater awareness and rigor about reporting accidents.

**Table 1:** Notifications of occupational accidents per year of notification, from 2012 to 2022

Year of notification	Notifications
Total	1,428,749
2012	75,481
2013	88,440
2014	83,495
2015	87,756
2016	85,242
2017	92,585
2018	100,404
2019	116,346
2020	176,859
2021	221,034
2022	301,107

Source: Sistema de Informação de Agravos de Notificação (SINAN) - Brazil Notifiable Disease Surveillance System.

This annual increase not only reflects a growing concern for safety in the workplace, but also highlights an urgent need to implement effective actions to prevent accidents and protect workers. A critical point appears to be the year 2020, with 176,859 notifications, marking a notable increase on previous years and anticipating the expressive growth recorded in 2021 and 2022. This data highlights the importance of more detailed analysis and proactive action in the workplace to mitigate risks and ensure workers’ health and safety.

The profile of occupational accident victims reveals significant differences between genders and age groups. Among men, the incidence is higher between 18-24 years old, while among women, accidents are more frequent between 30-34 years old. The most common injuries include cuts, lacerations, fractures, contusions, crushings, strains and sprains, among others.^12^

The practice of accident prevention, as presented in the book “Prática da prevenção de acidentes: ABC da segurança do trabalho,”^13^ highlights the importance of personal protective equipment (PPE), which is the only means capable of providing protection to the worker who is directly exposed to occupational risk. However, the use of PPE by workers is unfortunately scarce, which aggravates occupational accidents.

It is worth noting that occupational accidents contribute significantly to health costs in Brazil. According to information from the INSS, more than half of the benefits granted by the Social Security system (62.8%) refer to time off work due to occupational diseases and illnesses, highlighting the significant incidence of occupational accidents in the Brazilian social security scenario.^14^

Table 2 shows the evolution of occupational accident claims based on disability covering 2012 to 2022. The data reveals a concerning tendency for temporary partial disability temporary total disability permanent partial disability and permanent total disability cases to increase over time. In 2012, the number of cases of temporary disability was 39,412, and this figure grew progressively, reaching 118,272 in 2022. Similarly, permanent partial and permanent total disability have considerably increased over the years. In 2022, there was a significant increase in cases of permanent partial disability (4,803) and permanent total disability (327) compared to previous years.

**Table 2:** Evolution of cases of disability, per year of notification, from 2012 to 2022

Year of notification	Temporary disability	Permanent partial disability	Permanent total disability
Total	664,819	37151	3,433
2012	39,412	2,414	271
2013	45,212	2,708	292
2014	44,131	2,570	314
2015	47988	2,527	318
2016	48,772	2,580	299
2017	52,929	3,662	335
2018	56,055	4,513	347
2019	62,778	4,447	359
2020	65,807	3,149	271
2021	83,463	3,778	300
2022	118,272	4,803	327

Source: Sistema de Informação de Agravos de Notificação (SINAN) - Brazil Notifiable Disease Surveillance System.

These figures suggest not only an increase in the total number of accidents, but also the concerning severity of their consequences. This reinforces an urgent need to implement preventive measures and interventions in the workplace to reduce the prevalence and severity of accidents, with a view to protecting the health of workers. SINAN provided this information and is a reliable basis for analyzing and formulating occupational safety strategies.

Accident sickness benefit is a social security benefit granted by article 201, item I^15^ of the Constituição Federal (Brazilian Federal Constitution) and regulated by article 59 *et seq.* of Law 8,213/91.^1^ This benefit should be granted to injured workers, insured by the Social Security system, who are unable to work for more than 15 calendar days.

Table 3 presents a comprehensive overview of the evolution of cases of occupational accidents, according to the criteria of cure or death, from 2012 to 2022. The figures indicate a significant increase in cases of cure over the years, from 13,664 in 2012 to a significant total of 432,249 in 2022. This growth can be interpreted as a reflection of improvements in health care, safety protocols, and workplace interventions. However, it is crucial to note the growing cases of death from accidents, which also show an upward trend, from 1,957 in 2012 to 3,096 in 2022.

**Table 3:** Evolution of cases according to the criterion of cure or death caused by the accident, per year of notification, from 2012 to 2022

Year of notification	Cure	Death
Total	432,249	26,108
2012	13,664	1,957
2013	17,425	2,141
2014	16,889	2,138
2015	17759	2,050
2016	15,768	2,075
2017	17961	2,388
2018	19,221	2,412
2019	26,218	2,614
2020	75,400	2,276
2021	90,001	2,961
2022	121,943	3,096

Source: Sistema de Informação de Agravos de Notificação (SINAN) - Brazil Notifiable Disease Surveillance System.

Although the increase in deaths represents a relatively small percentage compared to the number of cure, it indicates a significant increase in the severity of occupational accidents, resulting in fatalities. These figures underscore the continued importance of implementing and reinforcing preventive and safety measures in the workplace to reduce not only the rate of accidents, but also their lethality, with a view to protecting the well-being of workers. Analysis of these figures is essential to guide the formulation of occupational safety policies and targeted interventions, with the aim to minimize the risks and adverse impacts on workers.

Over the years 2012 to 2022, the mortality rate due to occupational accidents showed annual variations. In 2012, this rate was approximately 2.59%, corresponding to 1,957 deaths out of a total of 75,481 notifications. In subsequent years, a variable trend was observed, with rates of 2.42%, 2.56%, and 2.33% in 2013, 2014, and 2015, respectively. In 2016, the rate rose to 2.43%, followed by an increase to 2.58% in 2017. In the following years, the mortality rate decreased, reaching 2.40% in 2018, 2.25% in 2019, and a significant decrease to 1.29% in 2020 and 1.34% in 2021. In 2022, the mortality rate was 1.03%. These variations reflect the complex dynamics affecting safety at work and suggest the continued importance of preventive policies to ensure a safe workplace and to protect workers’ lives.

Table 4 shows a detailed analysis of occupational accident notifications based on the levels of education of workers, from 2012 to 2022. The figures show significant patterns in notifications, highlighting the distribution of accidents according to level of education. In 2012, 738 notifications were recorded among illiterate workers, while those with primary education totaled 5,819 notifications. Over the years, a general upward trend in notifications at all levels of education is seen. In 2022, illiterate workers showed a substantial increase, reaching 1,344 notifications, in contrast to the 738 recorded in 2012. Notably, workers with secondary and higher education also experienced significant increases over the years, highlighting a need to consider different occupational safety strategies based on educational level.

**Table 4:** Notifications per level of education, from 2012 to 2022

Year of notification	Illiterate	Elementary school	High school	Higher education
Total	9,448	98,546	382,887	69,745
2012	738	5,819	15,223	1,578
2013	821	6,976	19,055	2,070
2014	774	6,501	18,045	1,991
2015	770	6,545	18,645	2,316
2016	639	6,353	19,596	2,611
2017	813	6,768	22,842	3,116
2018	896	7389	25,133	3,502
2019	896	8,810	31,453	4,588
2020	745	11,369	54,252	15,231
2021	1,012	13,917	65,168	15,297
2022	1,344	18,099	93,475	17445

Source: Sistema de Informação de Agravos de Notificação (SINAN) - Brazil Notifiable Disease Surveillance System.

This analysis highlights the importance of occupational safety policies which consider educational inequalities, with a view to implementing appropriate preventive measures to protect workers at different levels of education. A thorough grasp of these data can guide the formulation of strategies aimed at specific groups, contributing to the creation of safer workplaces and the promotion of occupational health throughout the workforce.

Table 5 shows an analysis of notifications of occupational accidents from 2012 to 2022, per region in Brazil. The results show significant variations in the geographical distribution of these accidents, pointing to patterns worthy of attention and study. In the baseline year of 2012, the Southeast region led the notifications, with 45,221 cases, followed by the Northeast region, which registered 8,682 cases. Over the years, there has been a notable change in this scenario. In 2022, the Southeast region maintained its prominent position, accounting for 104,502 cases, but the North region emerged significantly, taking second place with 43,072 notifications. The Northeast also stood out, registering 75,206 cases in 2021. This regional dynamic reflects the complexity of occupational safety issues, pointing to a need for specific and locally relevant approaches for each region. These figures not only highlight regional inequalities in the rate of occupational accidents, but also point to the importance of effective, regionally adapted occupational safety strategies.

**Table 5:** Notification per region, from 2012 to 2022

Year of notification	North	Northeast	Southeast	South	Midwest
Total	89,502	199,962	627,141	367,789	144,348
2012	5,265	8,682	45,221	7,924	8,389
2013	7,328	9,260	51,103	11,179	9,570
2014	5,806	9,355	47,835	10,453	10,046
2015	4,978	11,307	47,906	13,339	10,226
2016	4,947	9,780	46,738	13,458	10,319
2017	5,736	13,404	49,682	13,563	10,200
2018	6,153	15,434	50,561	15,616	12,640
2019	6,920	16,787	52,138	26,544	13,957
2020	11,463	31,060	56,249	64,929	13,158
2021	11,467	31,828	75,206	83,949	18,584
2022	19,439	43,072	104,502	106,835	27,259

Source: Sistema de Informação de Agravos de Notificação (SINAN) - Brazil Notifiable Disease Surveillance System.

According to information from the Instituto Brasileiro de Geografia e Estatística (IBGE, Brazilian Institute of Geography and Statistics), the Southeast region of Brazil is known for concentrating several industrial hubs, and is one of the most industrialized areas in the country.^16^ As a result, it leads most of the occupational accident rates in Brazil.

Table 6 provides a comprehensive overview of occupational accident notifications according to health care or not from 2012 to 2022. The figures reveal that the majority of notifications involved seeking health care, with a total of 1,294,035 cases, in contrast to 96,013 cases in which information about health care was missing or blank. The importance of health care following occupational accidents is emphasized by the increasing trend in seeking care over the years. In 2012, 71,171 cases had health care, and this number increased progressively, reaching 282,361 in 2022.

**Table 6:** Notifications per health care, from 2012 to 2022

Year of notification	Missing/blank	Yes	No
Total	96,013	1,294,035	38,701
2012	2,613	71,171	1,697
2013	2,589	83,547	2,304
2014	2,473	78,928	2,094
2015	3,477	82,071	2,208
2016	2,476	80,708	2,058
2017	3,956	86,518	2,111
2018	16,975	81,401	2,028
2019	17594	96,172	2,580
2020	21,415	148,479	6,965
2021	11,925	202,679	6,430
2022	10,520	282,361	8,226

Source: Sistema de Informação de Agravos de Notificação (SINAN) - Brazil Notifiable Disease Surveillance System.

Table 6 also shows that a great number of cases provided no clear information about health care, representing a gap in the comprehensive analysis of the impacts of occupational accidents. Health care plays a crucial role in preventing complications, effective recovery, and minimizing damage to workers’ health. These figures emphasize a need to reinforce the importance of health care following occupational accidents, promoting practices that ensure a rapid and adequate response and contributing to the preservation of workers’ health and well-being. The analysis of these figures underscores the importance of strategies that ensure accurate documentation of health care, providing a more complete and informed view of the nature and severity of occupational accidents and supporting ongoing efforts to improve safety conditions in the workplace.

## CONCLUSIONS

The results reveal an alarming scenario of occupational accidents in Brazil, particularly from 2012 to 2022. The steady increase in notifications, reaching a concerning peak of 301,107 cases in 2022, suggests an upward trend that demands immediate care. This growth can be attributed to various factors, from changes in working conditions to greater rigor in reporting accidents.

In this sense, an increase in notifications of occupational accidents over the last decade is also due to the expansion of RENAST and CERESTs actions, which promote mandatory notification of work-related illnesses and diseases. There has also been an increase in awareness among those responsible for mandatory reporting.

A detailed analysis of disabilities resulting from occupational accidents, shown in the evolution of the cases presented, highlights not only the increase in the overall number of accidents, but also the worryingly serious consequences. The significant increase in cases of temporary partial disability, temporary total disability, permanent partial disability, and permanent total disability, especially in 2022, points to an urgent need for preventive measures and interventions in the workplace to reduce the incidence and severity of accidents.

The consequences of accidents, assessed in terms of cure or death, offer a balanced perspective. The increase in cured cases over the years suggests improvements in health care and safety protocols, but the rising incidence of deaths due to accidents, from 1,957 in 2012 to 3,096 in 2022, highlights the urgent need to implement specific measures to reduce not only the rate, but also the lethality of occupational accidents.

A review of the annual mortality rate provides insights into the complex dynamics affecting safety at work. Although fluctuations have occurred over the years, the recent general downward trend in rates suggests that preventive efforts may be having a positive effect. However, the rate still remains high, indicating the continued importance of improved occupational safety policies.

Evaluation of notifications based on the level of education of workers reveals significant inequalities, highlighting a need for differentiated approaches to occupational safety based on the level of education. Regional data, meanwhile, shows considerable differences in the rate of occupational accidents in different regions of Brazil, underlining the complexity of occupational safety issues and a need for regionally adapted strategies.

Analysis of some or no health care following occupational accidents highlights a growing demand for health care, reflecting a greater awareness of health care. However, information gaps on health care highlight a need for more accurate documentation in order to provide a more complete comprehension of the impacts of accidents and guide appropriate prevention strategies.

These results jointly point to an urgent need for more robust interventions in occupational safety policies in Brazil. The growing rate of accidents, together with the severity of consequences, highlights the importance of preventive measures and specific strategies to address inequalities. Awareness of the need for health care is positive, but the gap in documentation highlights the importance of improving data collection systems. These findings should guide future studies and the formulation of effective policies and practices to protect the health and well-being of Brazilian workers.

Thus, based on these results, this study showed the time trend of occupational accidents in Brazil, grouped per region, from 2012 to 2022. It also investigated the number of accidents according to the level of education and age group of the professionals affected. The analysis also looked at the evolution of reported cases and the rate of health care, providing a comprehensive and detailed view of the dynamics of these events over a decade.
